# Role of Oxidative Stress as Key Regulator of Muscle Wasting during Cachexia

**DOI:** 10.1155/2018/2063179

**Published:** 2018-03-28

**Authors:** Johanna Ábrigo, Alvaro A. Elorza, Claudia A. Riedel, Cristian Vilos, Felipe Simon, Daniel Cabrera, Lisbell Estrada, Claudio Cabello-Verrugio

**Affiliations:** ^1^Departamento de Ciencias Biológicas, Facultad de Ciencias Biológicas, Universidad Andres Bello, Santiago, Chile; ^2^Millennium Institute of Immunology and Immunotherapy, Santiago, Chile; ^3^Centro de Investigaciones Biomédicas, Facultad de Ciencias Biológicas & Facultad de Medicina, Universidad Andres Bello, Santiago, Chile; ^4^Laboratory of Nanomedicine and Targeted Delivery, Center for Integrative Medicine and Innovative Science, Faculty of Medicine, and Center for Bioinformatics and Integrative Biology, Faculty of Biological Sciences, Universidad Andres Bello, Santiago, Chile; ^5^Center for the Development of Nanoscience and Nanotechnology (CEDENNA), Universidad de Santiago de Chile, Santiago, Chile; ^6^Departamento de Gastroenterología, Facultad de Medicina, Pontificia Universidad Católica de Chile, Santiago, Chile; ^7^Departamento de Ciencias Químicas y Biológicas, Facultad de Salud, Universidad Bernardo O'Higgins, Santiago, Chile; ^8^Centro Integrativo de Biología y Química Aplicada, Universidad Bernardo O'Higgins, Santiago, Chile

## Abstract

Skeletal muscle atrophy is a pathological condition mainly characterized by a loss of muscular mass and the contractile capacity of the skeletal muscle as a consequence of muscular weakness and decreased force generation. Cachexia is defined as a pathological condition secondary to illness characterized by the progressive loss of muscle mass with or without loss of fat mass and with concomitant diminution of muscle strength. The molecular mechanisms involved in cachexia include oxidative stress, protein synthesis/degradation imbalance, autophagy deregulation, increased myonuclear apoptosis, and mitochondrial dysfunction. Oxidative stress is one of the most common mechanisms of cachexia caused by different factors. It results in increased ROS levels, increased oxidation-dependent protein modification, and decreased antioxidant system functions. In this review, we will describe the importance of oxidative stress in skeletal muscles, its sources, and how it can regulate protein synthesis/degradation imbalance, autophagy deregulation, increased myonuclear apoptosis, and mitochondrial dysfunction involved in cachexia.

## 1. Introduction

Skeletal muscle atrophy is a pathological condition mainly characterized by a loss of muscular mass and the contractile capacity of skeletal muscle that produces muscular weakness and decreased force generation [1–6]. This pathological condition affects a large number of individuals and can be generated by several causes, including pathologic status and aging. Among the main causes are disuse, a state that can be produced by prolonged rest, immobilization, or hind-limb unloading [7–9]; denervation, which is characterized by alterations in neuromuscular connections produced under clinical conditions, such as trauma, diabetic neuropathy, degenerative disease, and spinal cord injury [10–16]; sepsis, an inflammatory syndrome produced mainly by bacterial infections [17–21]; sarcopenia, a physiological process of aging that decreases mobility and aggravates inflammatory diseases and other age-related diseases [22–28]; and chronic diseases that cause collateral damage in muscles by producing atrophic conditions termed cachexia [29–38], which will be the focus of this review.

## 2. Cachexia

Cachexia is defined as a pathological condition that is secondary to illness and characterized by the progressive loss of muscle mass with or without loss of fat mass [39]. Cachexia typically manifests in patients with chronic diseases such as cancer, diabetes, obesity, chronic obstructive pulmonary disease (COPD), chronic heart failure (CHF), chronic liver disease (CLD), and chronic kidney disease (CKD) [40], which affect the quality of life and survival of patients [41]. In addition to chronic illness, cachexia is associated with diseases that cause inflammation such as AIDS and sepsis [42]. The prevalence of cachexia is 1% of the total patient population, and it is severely increased among cancer (50–80%), AIDS (10–35%), CHF (5–15%), CKD (30–60%), and COPD (27–35%) patients [42–44].

**Figure 1:**
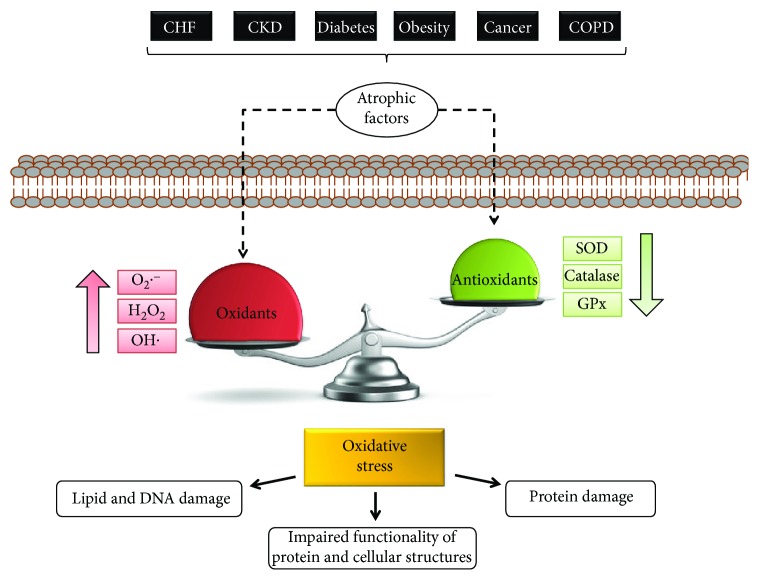
Oxidative stress in muscle is produced by an imbalance between oxidant and antioxidant species. Soluble atrophic factors produced by different diseases induce an imbalance of the oxidative state, increasing oxidant species such as O_2_·^−^, H_2_O_2_, and OH· and decreasing antioxidant species such as catalase, glutathione peroxidase (GPx), and superoxide dismutase (SOD). This imbalance is denominated as “oxidative stress” and produces oxidative damage in lipids, DNA, and proteins, impairing functionality of proteins and cellular structures.

Even though different types of diseases can induce cachexia, one important common feature of these conditions is alteration of the plasma levels of several soluble factors (termed “atrophic factors”), such as angiotensin II (Ang-II), transforming growth factor type beta (TGF-*β*), myostatin, glucocorticoids, tumor necrosis factor alpha (TNF-*α*), and interleukin 1 and 6 (IL-1 and IL-6) [45–54] (Figure 1). These molecules can modulate the different mechanisms involved in the loss of mass and function of skeletal muscle [3, 46, 48, 49, 53, 55–59].

### 2.1. Mechanisms Involved in the Generation and Development of Cachexia

**Figure 2:**
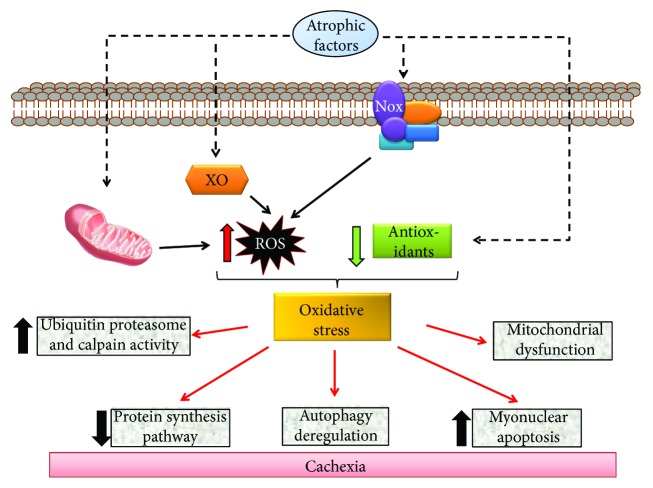
Molecular mechanisms involved in cachexia are modulated by oxidative stress. Atrophic factors can generate oxidative stress in skeletal muscle by the activation of different sources of reactive oxygen species, such as the mitochondria, xanthine oxidase (XO), and NADPH oxidase complex with Nox subunit, in addition to the decrease in antioxidant species. Oxidative stress is able to produce mitochondrial dysfunction, increase ubiquitin proteasome system activity, increase myonuclear apoptosis, decrease the protein synthesis pathway, and deregulate autophagy, all of which are involved in cachexia-skeletal muscle atrophy.

One of the main features of cachexia is the diminution of muscle strength. There are several molecular mechanisms and signaling pathways involved in cachexia that can explain this phenomenon: (i) oxidative stress, (ii) protein synthesis/degradation imbalance, (iii) autophagy deregulation, (iv) increased myonuclear apoptosis, and (v) mitochondrial dysfunction (Figure 2).

Oxidative stress is one of the most common mechanisms of different causes of cachexia, and two important characteristics of muscle in cachectic patients are increased ROS levels and oxidation-dependent protein modifications [60–62]. Additionally, oxidative stress can modulate other mechanisms involved in cachexia. In the following sections, we will describe the generation of oxidative stress, how oxidative stress regulates the aforementioned molecular mechanisms, and their roles in cachexia.

## 3. Oxidative Stress

Skeletal muscle is a tissue that continuously produces oxidant species such as reactive oxygen species (ROS) and reactive nitrogen species (RNS) (for details about RNS, see [63]), which are in balance with antioxidant mechanisms. The production of ROS species is a normal process in all cells (including skeletal muscle cells) in which signaling molecules regulate different pathways essential for cell viability [64, 65]. Skeletal muscle cells produce several types of ROS that differ in terms of origin, localization, stability, and reactivity [66]. The role of ROS in muscle can seem contradictory since they can act as signaling molecules in normal processes such as regeneration and repair [67] and promote mitochondrial biogenesis during exercise [68], but local sustained ROS levels may cause tissue injury due to oxidative damage [69].

The imbalance produced by an increase in oxidant species levels and/or a decrease in antioxidant species generates the loss of normal redox equilibrium in cells, a condition denominated as *oxidative stress*, which corresponds to redox status; can injure several cellular organelles, proteins, lipids, and membranes; and affects muscle function [70] (Figure 1).

The main features of oxidant and antioxidant species will be described in the following sections, and we will principally describe their participation and contribution to the generation of cachexia in patients with chronic diseases.

### 3.1. Types and Features of ROS

Superoxide anion (O_2_·^−^), hydrogen peroxide (H_2_O_2_), and hydroxyl radical (OH·) are the main ROS found in most tissues [64]. Several studies suggest that the major ROS produced in skeletal muscle fibers is O_2_·^−^ [71, 72], which is very labile and undergoes enzymatic or spontaneous dismutation by reduction to more stable species, such as H_2_O_2_. H_2_O_2_ is a nonradical weak oxidant with a relatively long half-life that can diffuse across cell membranes and therefore acts as an important intracellular signaling molecule [73, 74]. Additionally, H_2_O_2_ can generate OH· in the presence of active free iron ions or other transition metals, a process known as the Fenton reaction. OH· reacts immediately with any surrounding biomolecules, resulting in most of the deleterious effects associated with oxidative stress. In this context, considering that skeletal muscle contains 10–15% of total body iron—mainly in myoglobin and mitochondria—it could be especially sensitive to alterations due to oxidative stress. Thus, iron homeostasis can be considered a comodulator of ROS signaling and effects [75].

The main cellular macromolecules can be damaged by ROS. Cellular membranes can be damaged by the changes that produce OH· on lipids by attacking polyunsaturated fatty acid lipid residues and generating peroxyl radical [76]. DNA is affected because purine and pyrimidine bases and deoxyribose are damaged by OH [76]. OH· targets proteins by damaging their amino acid residues, such as lysine, arginine, histidine, proline, and threonine, causing the formation of protein carbonyls. In addition, the sulfhydryl group in amino acids undergoes irreversible oxidation [76].

### 3.2. Sources of ROS in Skeletal Muscle Cells

ROS in cells can be produced by different sources, such as mitochondria, sarcoplasmic reticulum, and sarcolemma. Additionally, the main enzymes involved in ROS generation under physiopathological conditions are nicotinamide adenine dinucleotide phosphate (NADPH) oxidase and xanthine oxidase (XO) (Figure 2).

The Nox protein family is composed of subunits of the NADPH oxidase enzyme complex that have catalytic and electron-transporting functions [77]. The Nox family consists of seven members, Nox1–5 and two dual oxidases (Duox), Duox1 and Duox2 [78]. Structurally, Nox isoforms contain FAD and NADPH binding sites, two heme molecules, and six transmembrane alpha helices with cytosolic N- and C-termini [78, 79]. Several proteins can interact with Nox isoforms. For example, Nox1–4 can bind to p22^phox^, while Nox1–2 can bind to small GTPases such as Rac. Nox2 can bind to p47^phox^ and p67^phox^ as well as the cytosolic protein p40^phox^ [78, 80]. Nox4 has been reported to bind to the polymerase (DNA-directed) delta-interacting protein 2 (PolDip2) [81]. NADPH oxidases are enzymes that serve a primary function in the production of superoxide/ROS. Nox1, Nox2, and Nox5 mainly produce O_2_·^−^, while Nox4 mainly produces H_2_O_2_ [82, 83]. Nox4 is constitutively active, and modulation of its expression may thus be a major activity regulator, whereas Nox1 can be activated by Nox activator 1 (NOXA1) protein, Nox2 can be activated by p67^phox^, and Nox5 can be activated by calmodulin [78, 79].

In skeletal muscle, the NADPH oxidase complex is reportedly located on transverse tubules (T-tubules), the sarcolemma, and the sarcoplasmic reticulum. In addition, skeletal muscle expresses only the Nox2 and Nox4 isoforms and partner proteins such as p22^phox^, p67^phox^, p47^phox^, and p40^phox^ [84, 85]. Interestingly, O_2_·^−^ generated from Nox has been implicated in progressive skeletal muscle damage [86]. Recent evidence demonstrated that NADPH oxidase overactivity leads to atrophy of glycolytic muscle in a rat model of heart failure (HF) [87]. Interestingly, the mechanism also involved the NF-*κ*B activation and increased p38 phosphorylation and was reduced by aerobic exercise training, suggesting that NADPH oxidase activity can be a good candidate for targeting and treating the muscle wasting [87].

Xanthine dehydrogenase (XDH), the most common form of xanthine oxidoreductase (XOR) in tissue, can be converted to xanthine oxidase (XO) via oxidation of sulfhydryl residues or proteolysis [88]. XO is an enzyme belonging to the molybdenum protein family with a homodimer structure and a molecule mass of 290 kDa. It contains two separate substrate-binding sites [88]. Functionally, XO causes oxidation of hypoxanthine to xanthine and then to uric acid [89, 90]. During reoxidation of XO, O_2_ acts as an electron acceptor, producing superoxide radical and hydrogen peroxide [91]. During these reactions, O_2_·^−^ and H_2_O_2_ are formed [91]. Spontaneously or under the influence of enzyme superoxide dismutase (SOD), O_2_·^−^ are transformed into H_2_O_2_ and O_2_ [88]. The conversion of XDH to XO is assumed to be required for radical generation and tissue injury, although some evidence suggests that XDH directly participates in O_2_·^−^ generation in ischemic tissue [92, 93]. In this context, it has been proposed that ischemia induces conversion of XDH into XO as well as production of hypoxanthine, which reacts with O_2_ during reperfusion and generates a high amount of superoxide radical from XO [94]. Early studies have suggested that ROS arising from XO plays an important role in the inflammatory response to physical eccentric contractions or high-intensity or long-lasting exercise as well as in injuries caused by ischemia-reperfusion processes [95, 96]. These studies are in agreement with those reporting the role of XO in muscle injury associated with exhaustive physical exercise [97–99]. In skeletal muscle, XO is localized mainly in the vascular endothelium [100]. The intake of enzyme inhibitors diminishes the release of O_2_·^−^ in the vessels of contracting muscles, which has proven to be effective for reducing muscle fatigue *in vivo* [101, 102]. Another study shows that suppression of XO activity by allopurinol may increase maximum isometric strength in the skeletal muscle of old mice [103]. In addition, administration of allopurinol and subsequent XO inhibition prevent muscular atrophy by inhibiting the p38 MAPK-atrogin-1 pathway and may have beneficial clinical effects, such as resistance against muscular atrophy in patients with permanent immobilization, sarcopenia, or cachexia [104, 105].

A third component that produces ROS in skeletal muscle is mitochondria. Skeletal muscle is a tissue that constantly demands ATP for energy production. ATP is generated via the activity of the mitochondrial electron transport chain (ETC) mainly at two sites: (i) complex I, where it is generated by auto-oxidation of the flavin mononucleotide from the NADH-dehydrogenase, and (ii) complex III, where its generation depends on auto-oxidation of unstable semiquinone, which is an intermediate of the Q-cycle reaction [106]. The ETC is located in the inner mitochondrial membrane. In this membrane, oxygen is consumed, resulting in the liberation of electrons that can quickly react with cellular proteins, resulting in their oxidation, or with molecules such as H_2_O or H_2_O_2_, generating more reactive molecules. Additionally, about 1–3% of the total oxygen utilized by the mitochondria is incompletely reduced and remains as ROS [107]. Compared with other tissues, skeletal muscle has a high number of mitochondria, and therefore, the contribution of this organelle to oxidative stress is very relevant.

### 3.3. Antioxidant Species in Skeletal Muscle

It is well known that skeletal muscle features high metabolic activity and oxidative capacity. Considering the importance of ROS production in skeletal muscle, the antioxidant system is essential for maintenance of cellular oxidative homeostasis. There are several antioxidant enzymes, including superoxide dismutase (SOD), catalase, and glutathione peroxidase (GPx) [108]. SOD has three isoforms: SOD1, which is located in the intracellular cytoplasmic compartment; SOD2, which is found in mitochondria; and SOD3, which is located in the extracellular matrix. This enzyme is a specific antioxidant for O_2_·^−^ and catalyzes the dismutation of O_2_·^−^ to H_2_O_2_ [108]. Some studies have indicated that mice lacking SOD1 lose muscle mass, suggesting that it plays a role in the maintenance of muscle fibers [109]. Catalase is present in cytoplasmic compartments and in mitochondria [110]. It catalyzes the conversion of H_2_O_2_ to H_2_O and O_2_ [111]. The enzymatic activity of catalase is higher in oxidative myofibers than in fast glycolytic fibers [112]. As an ROS scavenger, GPx has the same function, but with higher affinity for H_2_O_2_ than for catalase [108].

Five GPx isoforms have been described in mammals with different cellular localizations and substrate specificities. GPx1 is localized predominantly in the cytosol and somewhat in the mitochondrial matrix. GPx3 is present in the extracellular space [113]. GPx4 is a membrane-associated enzyme that is partly localized in the mitochondrial intermembrane space.

Studies have indicated that a decrease in antioxidant levels in response to diseases can lead to an imbalance in the redox state of the cell, causing oxidative damage [66, 114, 115] (Figure 1).

### 3.4. Oxidative Stress in Cachexia

Patients with chronic heart failure (CHF) or chronic kidney disease (CKD) develop cachexia associated with their pathologic status [116–118]. One of the main participants in this phenomenon is Ang-II, an endogenous peptide with atrophic activity in skeletal muscle. Patients with CHF and CKD have increased levels of circulating Ang-II [119–121]. Interestingly, Ang-II induces ROS production in skeletal muscle cells through its AT-1 receptor, as demonstrated by a study that found that losartan, an AT-1 receptor blocker, eliminates the oxidative effect of Ang-II [122]. Additionally, the atrophic effects mediated by Ang-II depend on ROS [123, 124]. In this context, Zhao et al. [125] and Cabello-Verrugio et al. [126] demonstrated that rats and mice infused with Ang-II have high ROS levels in skeletal muscle as well as major expression of gp91^phox^, a Nox subunit, suggesting that Nox increases ROS levels. Similar results were obtained in muscle cells incubated with Ang-II (i.e., they exhibited enhanced Nox activity) [124]. Moreover, the use of apocynin, a Nox inhibitor, blocks ROS production, suggesting that Ang-II increases ROS levels in skeletal muscle via a Nox-dependent mechanism [122]. Further, Ang-II promotes membrane mitochondrial depolarization, which increases mitochondrial ROS production, therefore contributing to oxidative stress in skeletal muscle [127]. Together, these results indicate that, in the presence of high levels of Ang-II, ROS is an important factor in the development of muscle atrophy in cachectic patients with chronic disease.

Patients with cancer cachexia have exhibited protein oxidation in skeletal muscle, suggesting the involvement of oxidative stress in cachexia [128]. In particular, patients with cancer present elevated ROS levels and decreased antioxidant levels in serum [66, 129]. They also have increased levels of mitochondrial uncoupling proteins (UCP) such as UCP2 and UCP3, which could lead to uncoupling of ETC and thus to the loss of mitochondrial membrane potential, increasing ROS production in mitochondria [130–133]. Additionally, cancer increases the levels of several proinflammatory cytokines involved in the pathogenesis of cachexia and oxidative damage, such as IL-1, IL-6, and TNF-*α* [134–137]. TNF-*α* induces ROS production by mitochondria and Nox activation [106, 138, 139]. Sullivan-Gunn et al. demonstrated that the expression of the Nox enzyme subunits Nox2, p40^phox^, and p67^phox^ was decreased in the muscle of mice with cancer cachexia, in spite of increased superoxide levels. However, these mice also exhibited decreased levels of antioxidant proteins such as SOD1, SOD2, and GPx [140], as reported previously [66, 141]. These results suggest that the development of oxidative stress in association with cancer-induced cachexia can be attributed, at least partially, to increased ROS levels and failure of the antioxidant systems that operate in muscle cells. Other evidence has indicated that inhibition of XO reduces skeletal muscle wasting and improves outcomes in a rat model of cancer cachexia, suggesting that other sources may contribute to oxidative stress [142].

## 4. Redox Regulation of Molecular Mechanisms of Cachexia

### 4.1. Imbalance in the Protein Synthesis/Degradation

All types of skeletal muscle atrophy are associated with a decrease in the levels of myofibrillar proteins, mainly myosin heavy chain, myosin light chain, and troponin, which are essential parts of the sarcomere structure [7, 39, 143]. The myosin proteins form a complex with actin and are responsible for muscle contraction [6]. In cachectic conditions, there is an imbalance in the degradation and/or synthesis of myofibrillar proteins, explaining their decreased levels. Under muscle atrophy conditions such as cachexia, the ubiquitin proteasome system (UPS) and calpains are the main mechanisms involved in the degradation of muscle proteins [144].

#### 4.1.1. The Ubiquitin Proteasome System

The UPS acts by the coordinated action of three enzymes: E1 (enzyme activator of ubiquitin), E2 (enzyme conjugator of ubiquitin), and E3 (ubiquitin ligase). All are involved in the labeling of specific proteins with ubiquitin (Ub) molecules. Ubiquitinated proteins are then degraded by proteasome 26S subunits [145]. E3 ubiquitin ligases are a family of enzymes that determine which protein will be ubiquitinated and degraded [1, 145]. In cachectic skeletal muscle, the levels of two E3 ubiquitin ligases are increased: MAFbx/atrogin-1 and MuRF-1. These muscle-specific enzymes target myofibrillar proteins, such as myosin, and factors involved in myogenesis, such as MyoD [145, 146]. Interestingly, our research and that of others have demonstrated that UPS is overactivated by soluble factors such as Ang-II and TGF-*β*1, which are increased during cachexia [45, 46, 48, 49, 147, 148].

UPS is the principal proteolytic mechanism described in skeletal muscle atrophy associated with chronic diseases. In pathological conditions, this pathway can be overactivated in multiple ways, including oxidative stress. Li et al. studied the effect of H_2_O_2_ on UPS markers in myotubes, showing that ubiquitin-conjugating activity is stimulated concomitant with an increase in the expression of E2 and E3 enzymes [149]. Additionally, a study by Russell et al. employing a murine model of cancer cachexia indicated that ubiquitin gene expression increases downstream Nox-generated ROS production, suggesting that Nox plays a role in cancer cachexia [124, 150] (Figure 2).

In chronic diseases, systemic increase of ROS can promote oxidative stress and alterations in peripheral tissues such as skeletal muscle, increasing the levels of proinflammatory transcription factors, such as nuclear factor kappa B (NF-*κ*B), that regulate specific UPS genes [60, 124]. In skeletal muscle, NF-*κ*B is activated and translocated to the nucleus to induce MuRF-1 expression [151]. Additionally, NF-*κ*B increases the expression of proinflammatory cytokines such as IL-6 and TNF-*α*, two important soluble factors involved in the development of skeletal muscle atrophy that increases ROS production and activate UPS, forming a positive feedback mechanism [50, 151–153].

These results indicate that, in skeletal muscle, ROS upregulates the expression of key components of UPS and increases their activity and that Nox participates in this phenomenon.

#### 4.1.2. Calpains

Calpains are Ca^2+^-activated proteases coded by 15 genes in humans that are involved in the selective cleavage of target proteins [154]. In skeletal muscle, calpain 1 (*μ*-calpain) and calpain 2 (m-calpain) participate in skeletal muscle atrophy [155]. Specifically, active calpains are able to cleave cytoskeletal proteins such as titin and nebulin, which are responsible for anchoring contractile proteins, as well as several kinases, phosphatases, and oxidized contractile proteins, such as actin and myosin [155, 156]. There is evidence that oxidative stress increases the expression of calpains in murine and human skeletal muscle cells [157, 158].

Studies have found that oxidative stress increases calpain activity in skeletal muscle cells [157, 158]. Specifically, H_2_O_2_ is able to increase calpain 1 activity in murine skeletal muscle cells and induce activation of calpain 1 and calpain 2 in human skeletal muscle cells [157, 158]. In line with these findings, antioxidant treatment of disused skeletal muscle has been found to prevent both oxidative stress and calpain 1 activation [159]. Together, these investigations confirm that oxidative stress in skeletal muscle can activate calpain.

The main regulators of calpain activity are cytosolic calcium and calpastatin, an endogenous calpain inhibitor [155, 160]. Thus, increased oxidative stress-dependent calpain activity is likely due to an increase in the cytosolic level of free calcium, which also depends on oxidative stress [158, 161, 162].

#### 4.1.3. Anabolic Pathways

Despite the fact that increased catabolism in skeletal muscle is the principal mechanism involved in the imbalance of protein content, reduced anabolism also contributes to this phenomenon. Induction of protein synthesis is determined by the Akt/mTOR (mammalian target of rapamycin) pathway and depends on insulin-like growth factor-1 receptor (IGFR), which can be activated by different factors, such as amino acids, insulin, and IGF-1. After IGFR binds to IGF-1, it is phosphorylated and activated, inducing activation of PI3K, which phosphorylates Akt and, consequently, mTOR, promoting protein synthesis [163]. Additionally, there is evidence that IGF-1 inhibits proteolysis in skeletal muscle by avoiding overactivation of UPS, suggesting regulation of both processes [164–166]. Previous reports have indicated that the circulating level of IGF-1 is reduced in patients with pathological conditions such as sepsis, cancer, and liver diseases [167–169]. Furthermore, soluble factors such as TNF-*α* and Ang-II act upstream of the IGF-1 pathway, inhibiting PI3K-Akt signaling and the downstream pathway. An example of this regulation involves the Forkhead box O (FoxO), a transcription factor normally phosphorylated by active PI3K-Akt/PKB that is kept inactive in the cytoplasm. When the synthesis pathway for TNF-*α* and Ang-II is inhibited, FoxO translocates to the nucleus and induces expression of the E3 ubiquitin ligases MAFbx/atrogin 1 and MuRF-1, increasing protein degradation [170].

Several factors, including ROS, are involved in the regulation of the PI3K-Akt pathway. Low ROS levels induce activation of the anabolic pathway, while high ROS levels inhibit it [171, 172] (Figure 2). Previous studies have established that Akt is a redox-sensitive protein that is activated in the presence of excess H_2_O_2_; however, this effect can be a consequence of indirect mechanisms such as oxidative inactivation of phosphatases or loss of feedback inhibition via MAPKs [173]. Increased ROS levels can induce protein oxidation in specific cysteine residues, inhibiting the activity of phosphorylases such as PKA that induce the activation of Akt [174]. The use of antioxidants such as *N*-acetyl cysteine (NAC) after oxidative stress stimulus prevents ROS increases and avoids inhibition of Akt activity [175], indicating that oxidation plays a role in this phenomenon. Additionally, inhibition of two important ROS sources, Nox and the mitochondrial ETC, activates Akt [175]. In skeletal muscle, ROS can be involved in the activation of metabolic effects by other signaling pathways independent of insulin, stimulating, for example, glucose transport during exercise, specifically during muscle contraction [176, 177].

### 4.2. Deregulation of Autophagy

The autophagy-lysosomal pathway is a normal mechanism that maintains cell homeostasis by removing old and damaged cellular components. This process eliminates portions of the cytoplasm, organelles, and protein aggregates in double-membrane vesicles, called autophagosomes, which are then fused with lysosomes for degradation [178]. Autophagy is often described as a five-step process: (1) induction, (2) expansion, (3) elongation and completion of autophagosomes, (4) fusion with lysosomes, and (5) degradation of proteins and organelles [63, 179]. Autophagy is induced by the formation of the pre-autophagosome structure, which occurs by activation of the ULK1 complex [179]. One of the main negative regulators of this step is mTORC1, and consequently all factors that prevent mTORC1 activation can promote autophagy [179]. The stage of expansion is characterized by the formation of phagophore, a fractional autophagosome membrane, and the recruitment of several Atg proteins, including the essential Atg6 (also called beclin-1) [179]. The elongation and completion of autophagosomes involve Atg genes (e.g., Atg5, Atg7, Atg8, and Atg12) [179]. During this stage, LC3B protein (Atg8) is posttranslationally modified from its inactive form (LC3I) to its active form (LC3II), which is a component of autophagosomes [180, 181]. Next, the autophagosome is fused with a lysosome, and the autophagosome's contents (i.e., cytosolic proteins and organelles) are transferred to lysosomal proteases (i.e., cathepsins B, D, and L) [179]. The fifth and final step of autophagy involves cathepsin-mediated degradation of proteins and organelles (i.e., the cargo) contained within the autophagosome [179–181].

Under pathological conditions, autophagy increases in association with muscle wasting induced by proatrophic stimuli, fasting, high-fat diet/insulin resistance, hypoxia, and exercise [182]. In addition, impaired autophagy has been reported in several myopathies [183–185]. Interestingly, a bidirectional relation between autophagy and oxidative stress has been reported, with some studies finding an increase in autophagy induced by ROS and other studies finding an increase in ROS induced by autophagy [182].

It has been demonstrated that, in patients with COPD, locomotor muscles feature increased autophagy [186, 187]. Recently, a study employing a murine model of sepsis induced by cecal ligation and perforation showed that limb muscles exhibit higher autophagy than do respiratory muscles [188]. Another recent study using an experimental model of CKD revealed a correlation between skeletal muscle oxidative stress, muscle catabolism, and autophagy, finding that inhibition of oxidative stress could improve muscle atrophy by enhancing mitophagy [189]. Moreover, a C26 cell-induced cancer model demonstrated that exercise increased autophagy flux, improving muscle homeostasis, probably due to the removal of damaged proteins and mitochondria [190].

Several studies have suggested that autophagy is activated by oxidative stress, but a study of expression of a mutant form of superoxide dismutase 1 (SOD1G93A) in skeletal muscle revealed a causal relation between oxidative stress, activation of autophagy, and muscle atrophy and weakness [191–194]. Although the mechanisms involved in the regulation of autophagy by ROS during skeletal muscle wasting are not yet known, studies have suggested that several signaling pathways participate in this regulation. Thus, it has been suggested that ROS can induce autophagy by regulating the activation of the PI3K/Akt/mTORC1 signaling pathway. A model of muscle atrophy by disuse demonstrated that ROS can inhibit Akt/mTOR signaling and consequently induce autophagy [195]. However, a skeletal muscle model employing dystrophic *mdx* mice revealed that Nox2-derived ROS can activate the Src/PI3K/Akt pathway and, subsequently, mTORC1, leading to autophagy inhibition [183].

Inactivation of PTEN (a phosphatase and tensin homolog deleted on chromosome 10) results in increased cellular PIP3 levels, activation of PI3K/Akt, and subsequent activation of autophagy [182]. One inhibitor of PTEN is oxidative stress [196, 197]. PTEN can also regulate ROS production, resulting in a feedback loop in which it has been suggested that Nox participates in [197]. While ROS has been shown to activate Akt through inhibition of PTEN in C2C12 myotubules, its role in regulating autophagy in skeletal muscle has not been directly assessed [196].

ROS-dependent regulation of autophagy may also occur through p38 MAPK. In skeletal muscle, the participation of p38 MAPK in autophagy was found in a model of muscle atrophy induced by sepsis [17]. The same model was used to demonstrate the involvement of ROS in p38 MAPK regulation of autophagy [198]. In other tissues, the p38 MAPK/p53 pathway has been shown to activate autophagy, but this pathway has not yet been evaluated in skeletal muscle [199, 200].

AMPK, a widely investigated indicator of cellular energy levels and regulator of muscle metabolism during exercise, may be another possible mechanism for redox regulation of autophagy in association with skeletal muscle wasting [201]. Alterations in redox balance have been shown to regulate AMPK activity [202]. Moreover, a study using C2C12 cells showed that, during nutrient deprivation and rapamycin treatment, there is an increase in mitochondria-derived ROS, which promotes skeletal muscle autophagy, and this effect is mediated in part by activation of AMPK and inhibition of Akt [194].

### 4.3. Myonuclear Apoptosis

Apoptosis is defined as programmed cell death. In skeletal muscle, this process is called myonuclear apoptosis and has distinctive characteristics compared to apoptosis of other tissues because muscle fibers are multinucleated cells. Myonuclear apoptosis involves elimination of the fiber segments that surround the apoptotic nucleus (known as the myonuclear domain), not the complete fiber [203–205].

The mechanisms involved in the generation of apoptotic nuclei have not been clearly elucidated. However, two principal signaling pathways are involved in apoptosis: extrinsic and intrinsic pathways. The extrinsic pathway is mediated by factors of the TNF family or Ang-II, which activate death receptors and induce activation of pro-caspase 8 by proteolytic cleavage. The intrinsic pathway is dependent on mitochondria and triggers an imbalance between antiapoptotic factors such as Bcl-2 (diminished) and apoptotic factors such as Bax (elevated) that might induce cytochrome *c* release and promote the formation of the mitochondrial transitory pore. Then, cytochrome *c* binds to apoptosis protease-activating factor-1 (Apaf-1) and pro-caspase 9 in the cytoplasm to form an apoptosome complex, which induces activation of caspase 9 (initiator caspase) [206]. Both the extrinsic and intrinsic pathways converge in the activation of effectors such as caspase 3. Caspase 3 activates endonuclease G, which triggers DNA fragmentation, degradation of genetic material by proteases, and posterior formation of apoptotic bodies eliminated by phagocytic cells.

Myonuclear apoptosis is increased in pathologies such as COPD, CHF, CKD, and obesity [38, 207–210]. Our group and others have found that cachectic muscle induced by Ang-II develops myonuclear apoptosis and that this is one of the main factors involved in overactivation of myonuclear apoptosis and the consequent increase in muscle weakness [45, 118, 211–213].

In other cell types, oxidative stress has been described as a potent inducer of cell death [214]. In an experimental model of cancer cachexia in which an XO inhibitor was used to reduce caspase-3 activity, Springer et al. showed that ROS production and proteasome activity decrease in skeletal muscle and consequently prevent body weight loss in animals [142]. Additionally, the mitochondrial apoptotic pathway is activated by a direct or indirect effect of ROS because increasing ROS can induce expression and mitochondrial translocation of the proapoptotic factor Bax, in turn inducing formation of the mitochondrial transition pore. Patients with cancer or CHF often present with hyperuricemia (incremented levels of uric acid), a condition in which XO activity is upregulated in the affected tissue and the systemic ROS level is increased [215]. Recently, studies employing a murine model of obesity induced by a high-fat diet (HFD) have shown that muscle weakness and protein degradation are accompanied by increased ROS levels and myonuclear apoptotic markers in muscle fibers [216].

### 4.4. Mitochondrial Dysfunction

Mitochondria play a key role in muscle physiology and metabolism. As mentioned throughout this review, mitochondria are the main producers of ATP and one of the main sources of ROS. However, other signaling intermediates such as calcium, NAD^+^/NADH, acetyl-CoA, and alpha-ketoglutarate are also produced/released to control muscle metabolism and epigenetics [217–219]. Mitochondrial function depends on the success of the mitochondrial life cycle, which involves mitochondrial biogenesis, remodeling through mitochondrial fusion and fission events called mitochondrial dynamics (MtDy), and degradation through a process called selective mitochondrial autophagy or mitophagy [220–223]. Any disruption of the mitochondrial life cycle will lead to mitochondrial dysfunction, which is characterized by low ATP levels and/or high ROS production [224, 225].

Superoxide (O_2_·^−^) is a byproduct of the ETC that can be converted to H_2_O_2_ by SOD2. As previously mentioned, O^2−^ and H_2_O_2_, which are both abundant in mitochondria, generate OH· (hydroxyl radical), which is the most reactive and harmful reactive radical for mitochondrial function. ROS will not only oxidize the respiratory complexes of ETC and mitochondrial DNA, among other macromolecules, but will also increase ROS production by damaged mitochondria, leading to a vicious cycle that ends in cell death due to apoptosis and/or necrosis [226, 227].

In addition to the antioxidant mechanisms previously described in this manuscript, mitochondria have more complex defense systems, including triple A proteases and mitochondrial unfolded protein response (mtUPR), which protect against cytotoxic protein aggregates and misfolded proteins, and the mitochondrial life cycle itself, which acts as a quality control system to eliminate old, dysfunctional, and depolarized mitochondria through mitophagy [225, 228–231].

The mitochondrial life cycle and defense systems are both defective in cachectic conditions, negatively impacting mitochondrial function. As previously reported, mitochondrial biogenesis, mitochondrial dynamics, and mitophagy are defective in skeletal and cardiac muscle cells with altered mitochondrial content and morphology; disruption of mitochondrial fusion and exacerbation of mitochondrial fission; altered mitophagy; reduced ETC activity; increased ROS generation; and proneness to apoptosis and mPTP opening [232, 233]. At the level of mitochondrial content, there is a reduction in the expression of PGC1-alpha, the master regulator of mitochondrial biogenesis; nuclear receptor factor 1 and transcription factor A, both of which control nuclear and mitochondrial gene expression for proper mitochondrial function; and SIRT1, a deacetylase that controls PGC1-alpha activity [232, 233]. At the level of mitochondrial dynamics, expression of the fusion proteins MFN1 and MFN2 reduces, and the level of the FIS1 and DRP1 fission proteins is increased [232, 233]. In addition, mitophagy, defined by expression of the LC3, PARKIN, PINK, and Atg5 markers, increases. However, there are some controversial points about mitophagy, which will be discussed later. Finally, at the level of the ETC, the respiratory complexes cytochrome *c* oxidase (complex IV) and cytochrome *bc*_1_ (complex III) and the mobile component of cytochrome *c* showed reduced expression. A similar result was observed for the enzyme citrate synthase that forms part of Krebs cycle [232, 233].

It has been recently shown that mitochondrial biogenesis, mitochondrial dynamics, and mitophagy are interconnected. This means that there is a perfect balance between the need for mitochondrial dynamics in mitophagy and the need for mitophagy in mitochondrial biogenesis [234–239]. Mitophagy is essential for mitochondrial turnover to maintain a healthy mitochondria population, control the amount of cellular ROS, and eliminate damaged and ROS-producing mitochondria. Thus, mitophagy failure is associated with an accumulation of dysfunctional mitochondria and decreased mitochondrial biogenesis. Several studies performed in cachectic muscle have reported increased mitophagy indicated by the expression of mitophagy markers [232]. However, other studies have reported reduced mitophagy in patients with cancer cachexia [224]. Given these conflicting findings, it is important to consider mitophagy in terms of flux. Diminished mitophagic flux will cause accumulation of mitophagic markers and damaged mitochondria and decreased mitochondrial biogenesis, generating a pool of dysfunctional mitochondria in accordance with the pathology of cachexia.

## 5. Conclusions

As mentioned in this review, cachexia is a pathological condition that affects skeletal muscle and leads to weakness and loss of strength and muscle mass. This condition is secondary to other pathologies that affect other tissues and is characterized by the participation of secreted soluble factors that produce an atrophic effect in skeletal muscle.

We know that different mechanisms are involved in the development of skeletal muscle atrophy, such as UPS overactivation, protein synthesis pathway diminution, autophagy deregulation, increased myonuclear apoptosis, and oxidative stress, which are activated depending on the stimuli. In this review, we have shown that, even though each mechanism can act independently and play an important role in muscle weakness, the mechanisms are interconnected. In particular, we emphasized oxidative stress as an atrophic mechanism that affects the other mentioned mechanisms. We highlighted the importance of redox state regulation in muscle cells in order to maintain homeostasis and the deleterious effects produced when this balance is lost. In conclusion, although all these mechanisms can generate harmful effects in muscle through different pathways, oxidative stress modulates all of them and can produce a more harmful effect or accelerate muscle damage. Therefore, reduction or prevention of oxidative imbalance in muscle is of vital importance.

## Abbreviations

AIDS: Acquired immune deficiency syndrome
AMP: Adenosine monophosphate
AMPK: AMP-activated protein kinase
Ang-II: Angiotensin II
Apaf-1: Apoptotic protease-activating factor-1
AT-1: Angiotensin II receptor type 1
Atg: Autophagy-related gene
ATP: Adenosine triphosphate
CHF: Chronic heart failure
CKD: Chronic kidney disease
CLD: Chronic liver disease
COPD: Chronic obstructive pulmonary disease
DRP1: Dynamin-related protein
Duox: Dual oxidases
E1: Enzyme activator of ubiquitin
E2: Enzyme conjugator of ubiquitin
E3: Ubiquitin ligase
ETC: Electron transport chain
FAD: Flavin adenine dinucleotide
FIS1: Mitochondrial fission 1 protein
FoxO: Forkhead box O
GPx: Glutathione peroxidase
GTPase: Guanosine triphosphatase
HFD: High-fat diet
H_2_O_2_: Hydrogen peroxide
IL-1: Interleukin 1
IL-6: Interleukin 6
IGF-1: Insulin-like growth factor-1
IGFR: Insulin-like growth factor-1 receptor
MAFbx: Muscle atrophy F-box
MAPK: Mitogen-activated protein kinases
MFN1: Mitofusin 1
mPTP: Mitochondrial permeability transition pore
MtDy: Mitochondrial dynamics
mtUPR: Mitochondrial unfolded protein response
MuRF-1: Muscle RING finger 1
MyoD: Myogenic differentiation factor
NAC: *N*-Acetyl cysteine
NADPH: Nicotinamide adenine dinucleotide phosphate
NF-*κ*B: Nuclear factor kappa B
NFR1: Nuclear receptor factor 1
Nox: NADPH oxidase subunity
NOXA1: Nox activator 1
O_2_·^−^: Superoxide anion
OH·: Hydroxyl radical
PGC1-alpha: Peroxisome proliferator-activated receptor gamma coactivator 1-alpha
PI3K: Phosphoinositide 3-kinase
PINK: PTEN-induced putative kinase
PKA: Protein kinase A
PolDip2: Polymerase (DNA-directed) delta-interacting protein 2
PTEN: Phosphatase and tensin homolog deleted on chromosome 10
RNS: Reactive nitrogen species
ROS: Reactive oxygen species
SIRT1: Sirtuin 1
SOD: Superoxide dismutase
Src: Proto-oncogene tyrosine-protein kinase
TFAM: Transcription factor A, mitochondrial
TGF-*β*: Transforming growth factor type beta
TNF-*α*: Tumor necrosis factor-alpha
UPS: Ubiquitin proteasome system
UCP: Uncoupling proteins
ULK1: Unc-51 like autophagy activating kinase
XDH: Xanthine dehydrogenase
XO: Xanthine oxidase
XOR: Xanthine oxidoreductase.
